# Legal Responsibility

**Published:** 1856-05

**Authors:** 


*Legal Responsibility.*—Judge Minot, of Pennsylvania, has laid down the following rules of law, as applicable to physicians: 1. The medical man engages that he possesses a reasonable degree of skill, such as is ordinarily possessed by a profession generally. 2. He engages to exercise that skill, with reasonable care and diligence. 3. He engages to exercise his best judgment, *but is not responsible for a mistake of judgment.* Beyond this, the defendant is not responsible. The patient himself must be responsible for all else; if he desires the highest degree of skill and care, he must secure it himself. 4. It is a rule of law that a medical practitioner never insures the result. These are received in general as sound views, and such as will govern every enlightened court. There could scarcely be a greater absurdity, than to require physicians and surgeons to insure the result, when they can in no case control all parts of the treatment. Few serious cases are carried through a single day, and many not a single hour, without a violation of instructions, on the part of nurses and attendants. —*Peninsular Journal of Medicine and Collateral Sciences.*

